# Effect of Three Antioxidants on the Shear Bond Strength of Composite Resin to Bleached Enamel: An In Vitro Study

**DOI:** 10.7759/cureus.114722

**Published:** 2026-08-18

**Authors:** Arpit Aggarwal, Navjot S Khurana, Jagvinder S Mann, Jaspreet Kaur, Shivam Garg, Geesiyes Yadav

**Affiliations:** 1 Department of Conservative Dentistry and Endodontics, Government Dental College and Hospital, Patiala, IND

**Keywords:** antioxidants, bleaching, composite resin, proanthocyanidin, selenium, shear bond strength, sodium ascorbate

## Abstract

Introduction

Vital tooth bleaching is a widely accepted minimally invasive treatment for the management of tooth discoloration. However, residual oxygen radicals generated during bleaching adversely affect the polymerization of resin-based restorative materials, resulting in reduced bond strength when adhesive procedures are performed immediately after bleaching. Antioxidant agents have been proposed as a means of neutralizing residual free radicals and restoring bonding effectiveness.

Objective

The objective of this study is to compare the effects of 6.5% proanthocyanidin, 10% sodium ascorbate, and 10% selenium on the shear bond strength (SBS) of composite resin bonded to enamel following in-office bleaching.

Materials and methods

Seventy-five extracted permanent maxillary anterior teeth were selected and subjected to in-office bleaching using 37.5% hydrogen peroxide. Specimens were randomly allocated into five groups (n = 15): immediate bonding after bleaching, delayed bonding after three weeks, 6.5% proanthocyanidin treatment, 10% sodium ascorbate treatment, and 10% selenium treatment. Following surface treatment, enamel specimens were etched with 37% phosphoric acid, bonded using Adper™ Single Bond 2 (3M ESPE, St. Paul, MN, USA), and restored with Filtek Z250 XT (3M ESPE, St. Paul, MN, USA) composite resin. SBS was evaluated using a universal testing machine (UTM) at a crosshead speed of 0.5 mm/min. Data were analyzed using one-way analysis of variance (ANOVA) and Tukey’s post hoc test, with statistical significance set at p < 0.05.

Results

The highest mean SBS was observed in the proanthocyanidin group (45.52 ± 1.37 MPa), followed by sodium ascorbate (31.11 ± 1.98 MPa), delayed bonding (30.39 ± 1.51 MPa), selenium (15.95 ± 0.82 MPa), and immediate bonding (11.17 ± 1.66 MPa). The ANOVA demonstrated marked differences across the experimental groups (F = 1209.846, p < 0.001). Proanthocyanidin exhibited significantly higher SBS than all other groups.

Conclusion

Application of antioxidants after bleaching significantly improved the bond strength of composite resin to enamel. Among the tested antioxidants, 6.5% proanthocyanidin demonstrated the greatest efficacy in restoring SBS, followed by sodium ascorbate. Selenium exhibited comparatively limited effectiveness. Proanthocyanidin may serve as a clinically useful adjunct for immediate restorative procedures following bleaching.

## Introduction

Tooth discoloration is a common esthetic concern that can adversely affect an individual's self-esteem, interpersonal interactions, and overall well-being. In modern dentistry, increasing patient awareness and demand for esthetic treatments have led to a substantial rise in the utilization of tooth-whitening procedures [1]. Among the available treatment options, dental bleaching is considered one of the most conservative, cost-effective, and minimally invasive approaches for managing both intrinsic and extrinsic tooth discoloration.

Vital bleaching is routinely performed using hydrogen peroxide- or carbamide peroxide-based agents. These materials release reactive oxygen species capable of penetrating enamel and dentin and oxidizing chromogenic compounds responsible for tooth discoloration [2]. As a result, bleaching effectively improves tooth shade while preserving natural tooth structure.

Despite its clinical success, bleaching has been associated with several alterations in dental hard tissues. Previous studies have reported changes in enamel surface morphology, mineral content, microhardness, and permeability following exposure to peroxide-based bleaching agents [3]. More significantly, oxygen molecules and peroxide-related free radicals retained within the enamel following the bleaching procedure can inhibit the polymerization of adhesive resin materials by disrupting their free-radical curing mechanism. Consequently, immediate restorative procedures performed after bleaching often exhibit significantly reduced bond strength compared with unbleached enamel.

The conventional approach for overcoming this reduction in bond strength involves postponing adhesive procedures for one to three weeks, allowing residual oxygen to dissipate naturally from the enamel surface. Although effective, delayed bonding may not always be clinically feasible, particularly in patients seeking immediate esthetic rehabilitation or requiring replacement of existing restorations following bleaching treatment [4].

To overcome this limitation, several antioxidant agents have been investigated as chairside interventions capable of neutralizing residual free radicals and restoring the redox potential of bleached enamel [5]. By eliminating residual oxidizing species, antioxidants facilitate normal polymerization of adhesive resins and improve the bond strength of composite restorations.

Among the antioxidants evaluated in restorative dentistry, sodium ascorbate has received considerable attention because of its potent reducing activity and biocompatibility. Proanthocyanidin, a naturally occurring polyphenolic compound derived from grape seed extract, possesses exceptional free-radical scavenging ability and has demonstrated promising results in restoring bond strength following bleaching. Selenium, an essential trace element involved in antioxidant defense mechanisms through glutathione peroxidase activity, has recently emerged as a potential alternative antioxidant for dental applications [6].

Although several studies have independently evaluated these antioxidants, direct comparisons among sodium ascorbate, proanthocyanidin, and selenium under standardized bleaching conditions remain limited. Therefore, the present in vitro study was undertaken to compare the effectiveness of 6.5% grape seed extract containing proanthocyanidin, 10% sodium ascorbate, and 10% selenium in restoring the shear bond strength (SBS) of composite resin to enamel following bleaching with 37.5% hydrogen peroxide.

## Materials and methods

Study design 

This in vitro experimental study was conducted in the Department of Conservative Dentistry and Endodontics, Government Dental College and Hospital, Patiala, India.

Sample selection and preparation

**Figure 1:**
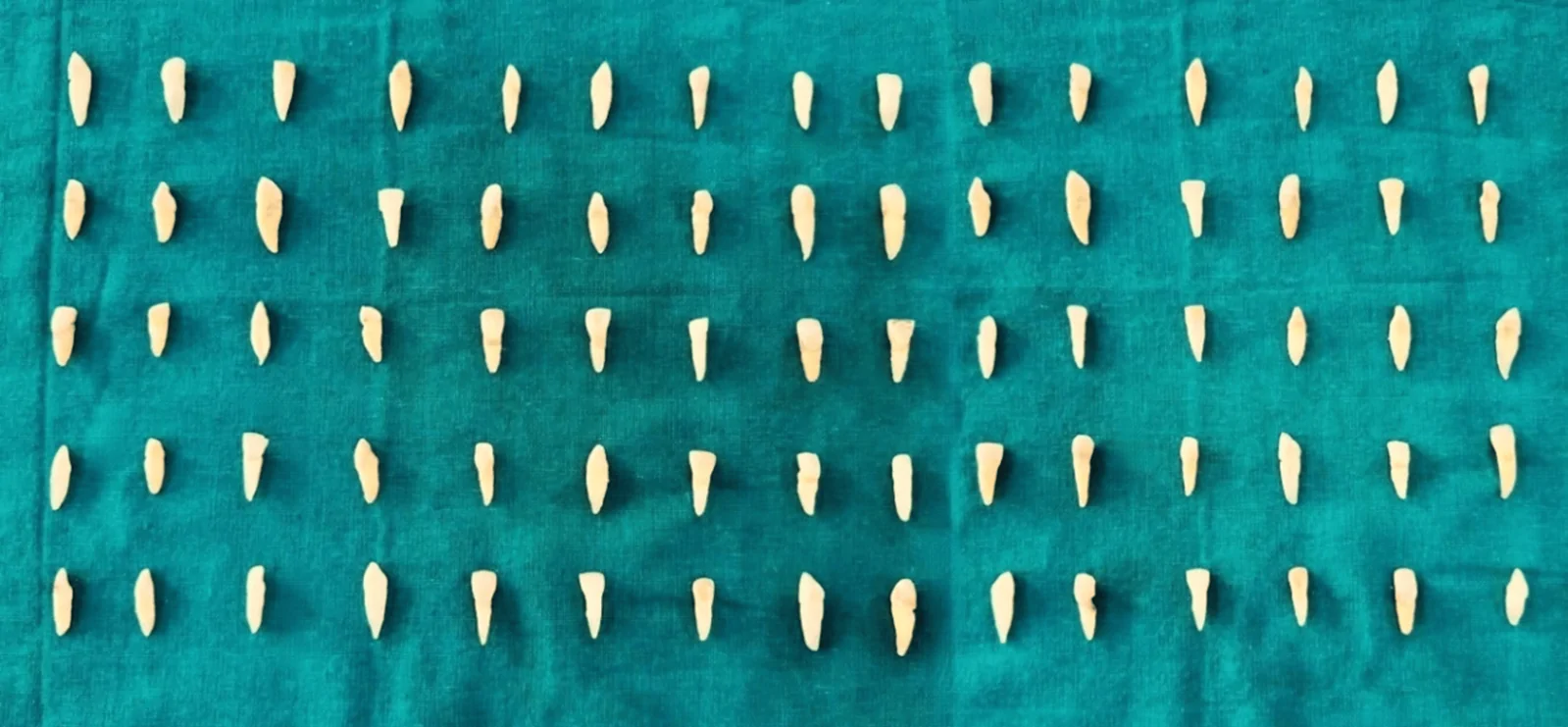
Seventy-five extracted maxillary anterior teeth

A total of 75 freshly extracted, non-carious permanent maxillary anterior teeth extracted for periodontal reasons were included in the study as shown in Figure 1. Teeth with caries, restorations, cracks, developmental defects, fluorosis, hypoplasia, or structural abnormalities were excluded.

Following extraction, soft tissue remnants and calculus deposits were removed using an ultrasonic scaler. The teeth were disinfected in 0.5% sodium hypochlorite solution, cleaned with a pumice-water slurry, rinsed thoroughly with distilled water, and stored in normal saline at room temperature until use.

Each tooth was embedded in a self-cure acrylic resin block up to the cementoenamel junction (CEJ) with the labial surface exposed. To obtain a standardized bonding surface, the labial enamel was flattened using 600-grit silicon carbide abrasive paper under continuous water irrigation.

Bleaching procedure

All specimens were subjected to in-office bleaching using 37.5% hydrogen peroxide gel (Pola Office+, SDI Limited, Bayswater, Australia). The bleaching agent was applied to the labial enamel surface in accordance with the manufacturer's instructions. Four consecutive applications were performed, each for 10 minutes, resulting in a total bleaching time of 40 minutes. Following bleaching, the specimens were thoroughly rinsed with distilled water and air dried.

Group allocation

After bleaching, specimens were randomly allocated into five groups (n = 15 per group) using a computer-generated randomization sequence: Group 1, immediate bonding after bleaching; Group 2, delayed bonding after three weeks; Group 3, treatment with 6.5% grape seed extract containing proanthocyanidin; Group 4, treatment with 10% sodium ascorbate; and Group 5, treatment with 10% selenium.

Preparation and application of antioxidants

The antioxidant solutions were freshly prepared immediately before application.

Group 3: Proanthocyanidin

**Figure 2:**
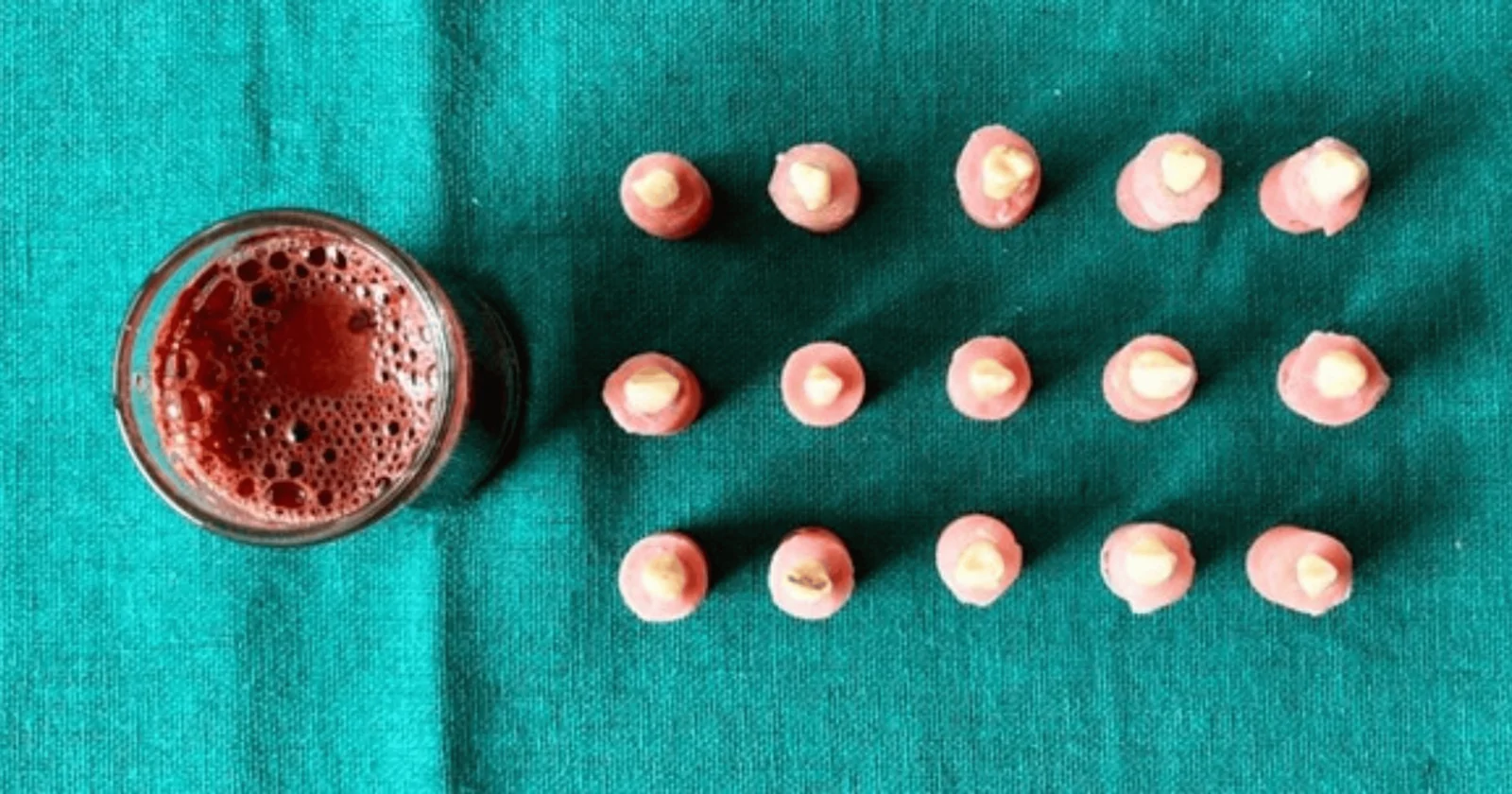
Preparation of 6.5% grape seed extract containing proanthocyanidin solution

A 6.5% grape seed extract containing proanthocyanidin solution was prepared by dissolving 6.5 g of grape seed extract powder in 100 mL of distilled water as shown in Figure 2.

Group 4: Sodium Ascorbate

**Figure 3:**
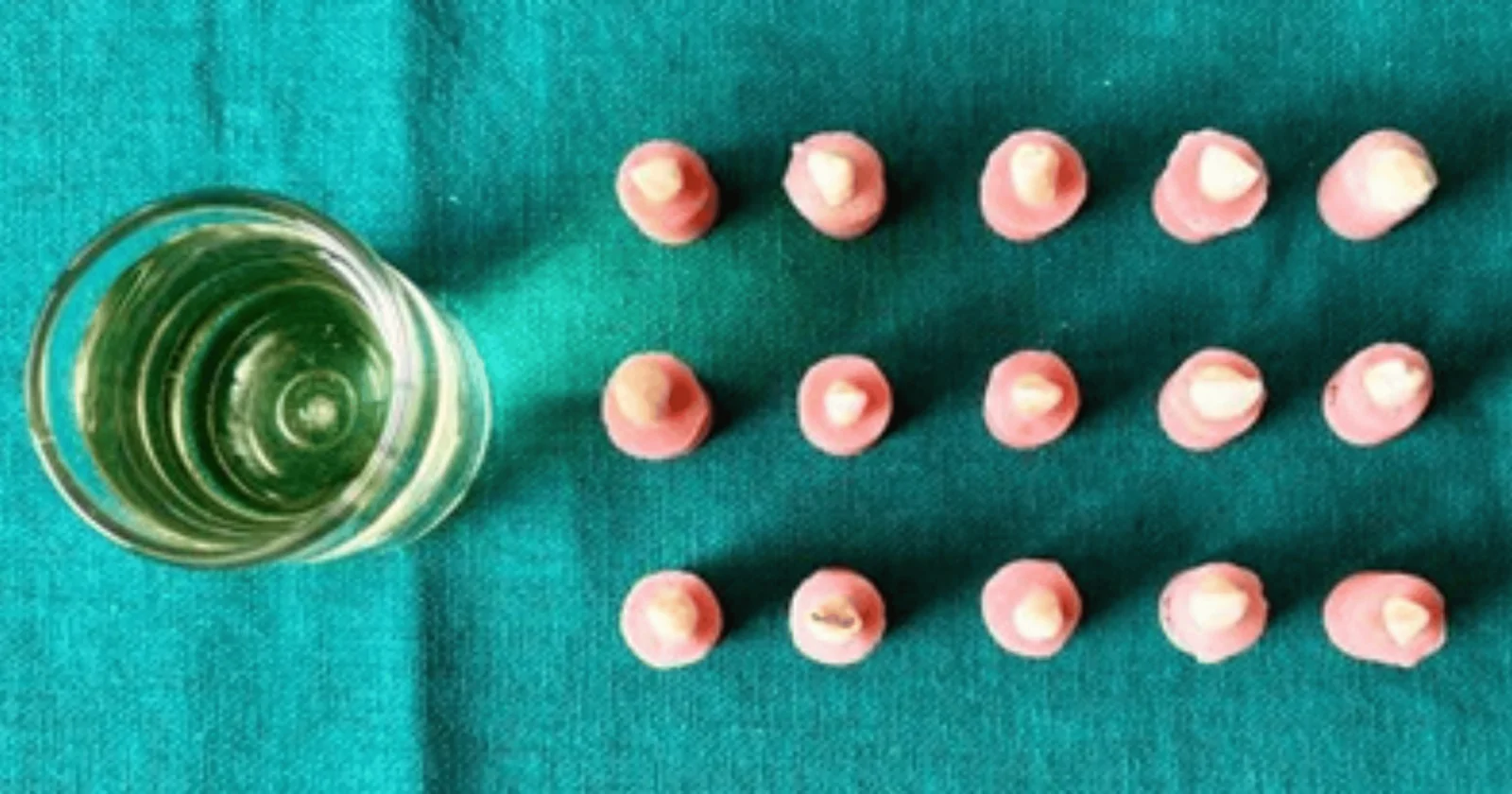
Preparation of 10% sodium ascorbate solution

A 10% sodium ascorbate solution was prepared by dissolving 10 g of sodium ascorbate powder in 100 mL of distilled water as shown in Figure 3.

Group 5: Selenium

**Figure 4:**
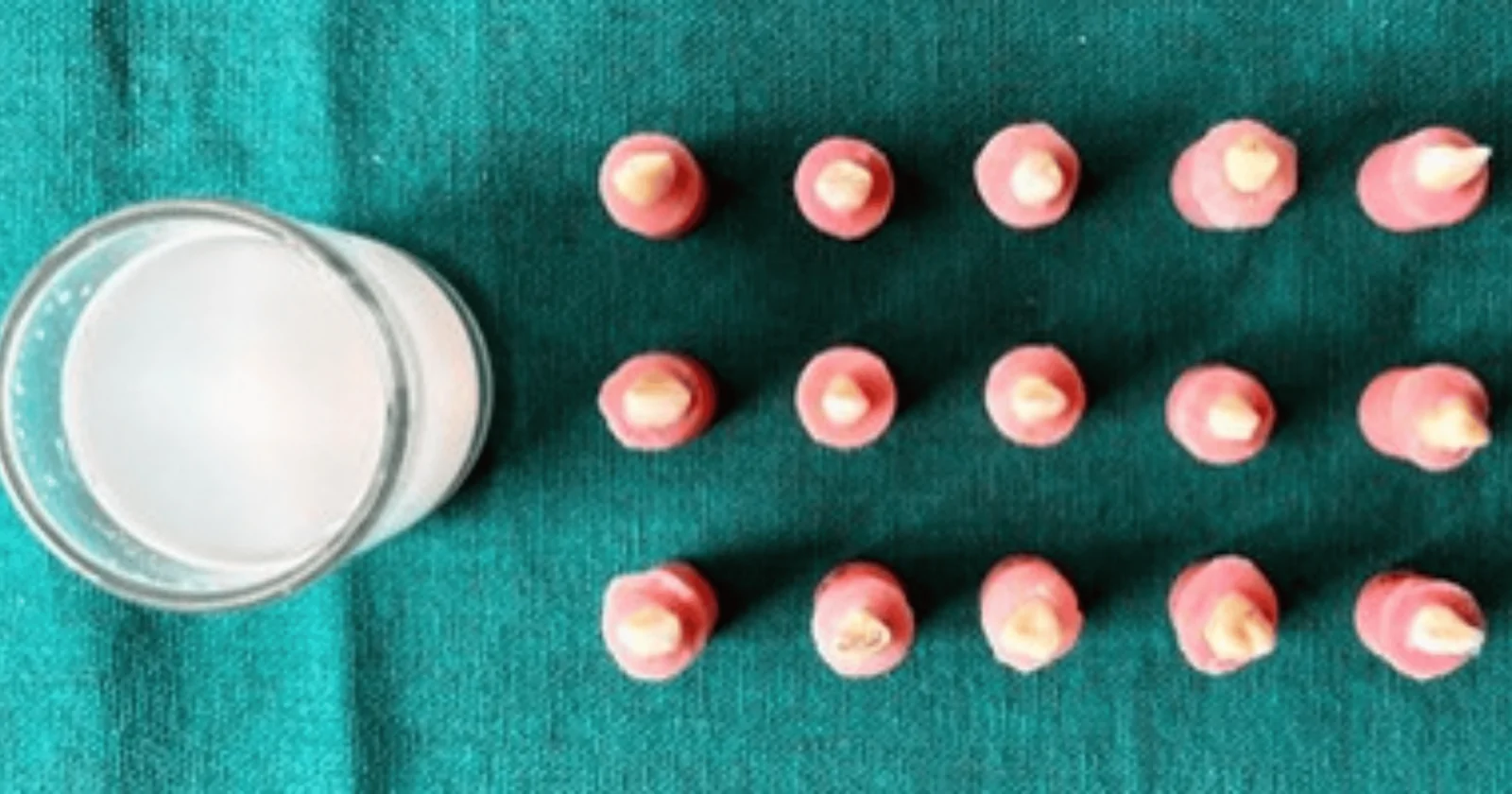
Preparation of 10% selenium solution

A 10% selenium solution was formulated by dissolving 10 g of selenium powder in 100 mL of deionized distilled water as shown in Figure 4.

The antioxidant solutions were applied to the enamel surfaces for 10 minutes with intermittent replenishment to ensure continuous surface contact. Following application, specimens were rinsed thoroughly with distilled water and gently air-dried.

Bonding procedure

The enamel surfaces were etched with 37% phosphoric acid gel (Scotchbond Multipurpose Etchant, 3M ESPE, St. Paul, MN, USA) for 30 seconds, rinsed with water for 15 seconds, and gently air-dried.

A bonding agent (Adper Single Bond 2, 3M ESPE, St. Paul, MN, USA) was applied according to the manufacturer's instructions and light-cured for 20 seconds using an LED curing unit.

A cylindrical Teflon mold with an internal diameter of 4 mm and a height of 2 mm was positioned on the enamel surface. Composite resin (Filtek Z250 XT, 3M ESPE, St. Paul, MN, USA) was placed in a single increment and light-cured for 20 seconds. After polymerization, the mold was carefully removed, leaving a standardized composite cylinder bonded to the enamel surface.

SBS testing

**Figure 5:**
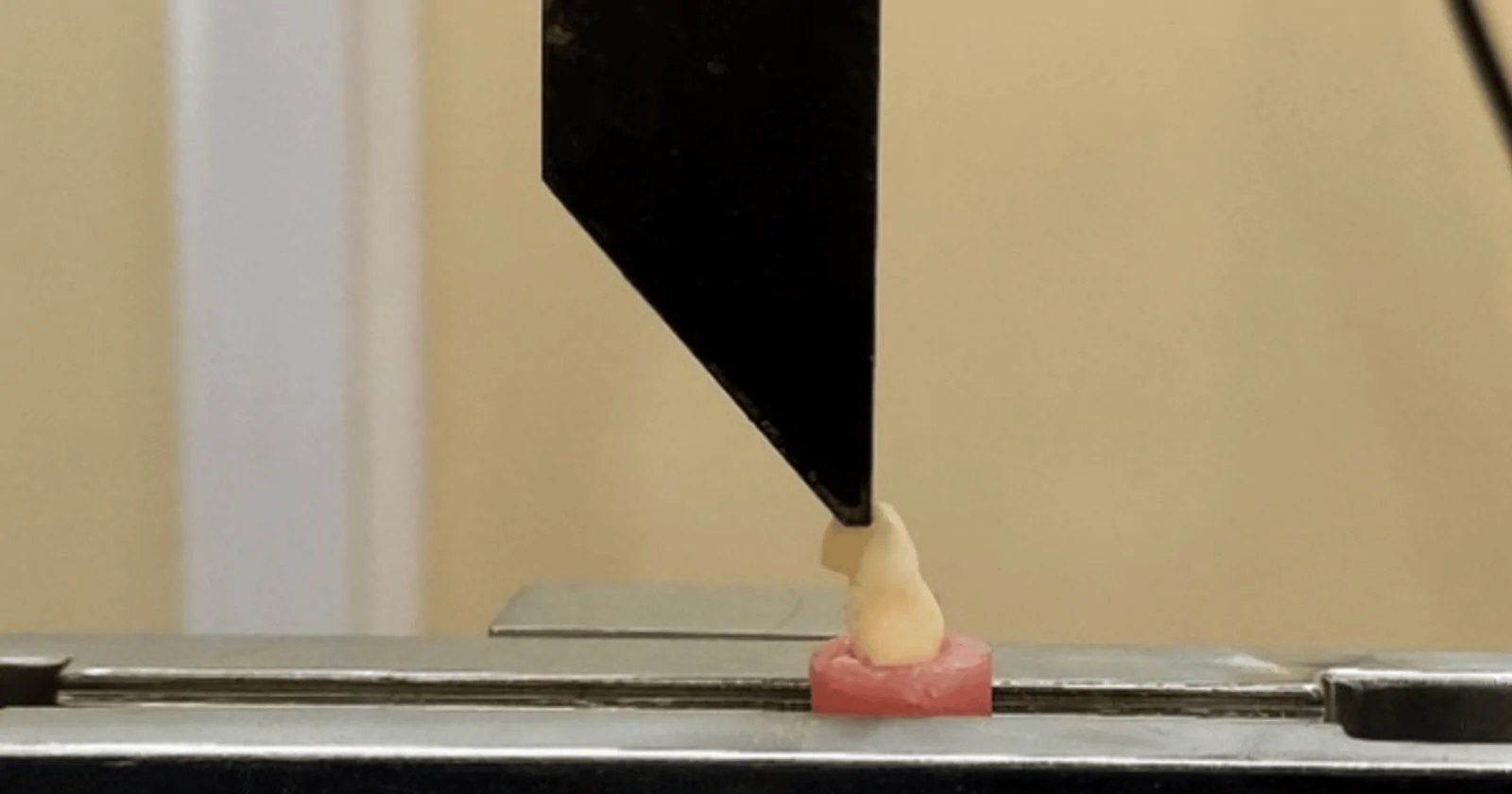
Shear bond strength testing under universal testing machine

All specimens were subjected to SBS testing using a universal testing machine (UTM). A knife-edge loading blade was positioned parallel to the tooth surface at the enamel-composite interface, as shown in Figure 5.

Load was applied at a crosshead speed of 0.5 mm/min until bond failure occurred. The maximum force required to debond the composite cylinder was recorded in Newtons (N).

SBS was calculated in megapascals (MPa) using the following formula: $\mathrm{SBS\ (MPa)} = \frac{\mathrm{Force\ at\ Failure\ (N)}}{\mathrm{Bonding\ Area\ (mm^2)}}$, where bonding area = πr², and r represents the radius of the composite cylinder.

Statistical analysis

Descriptive statistics including mean, standard deviation, minimum value, and maximum value were calculated for each group. Intergroup comparisons were performed using one-way analysis of variance (ANOVA). Pairwise comparisons between groups were carried out using Tukey's post hoc Honestly Significant Difference (HSD) test. A p-value of less than 0.05 was considered statistically significant.

## Results

Descriptive analysis

**Figure 6:**
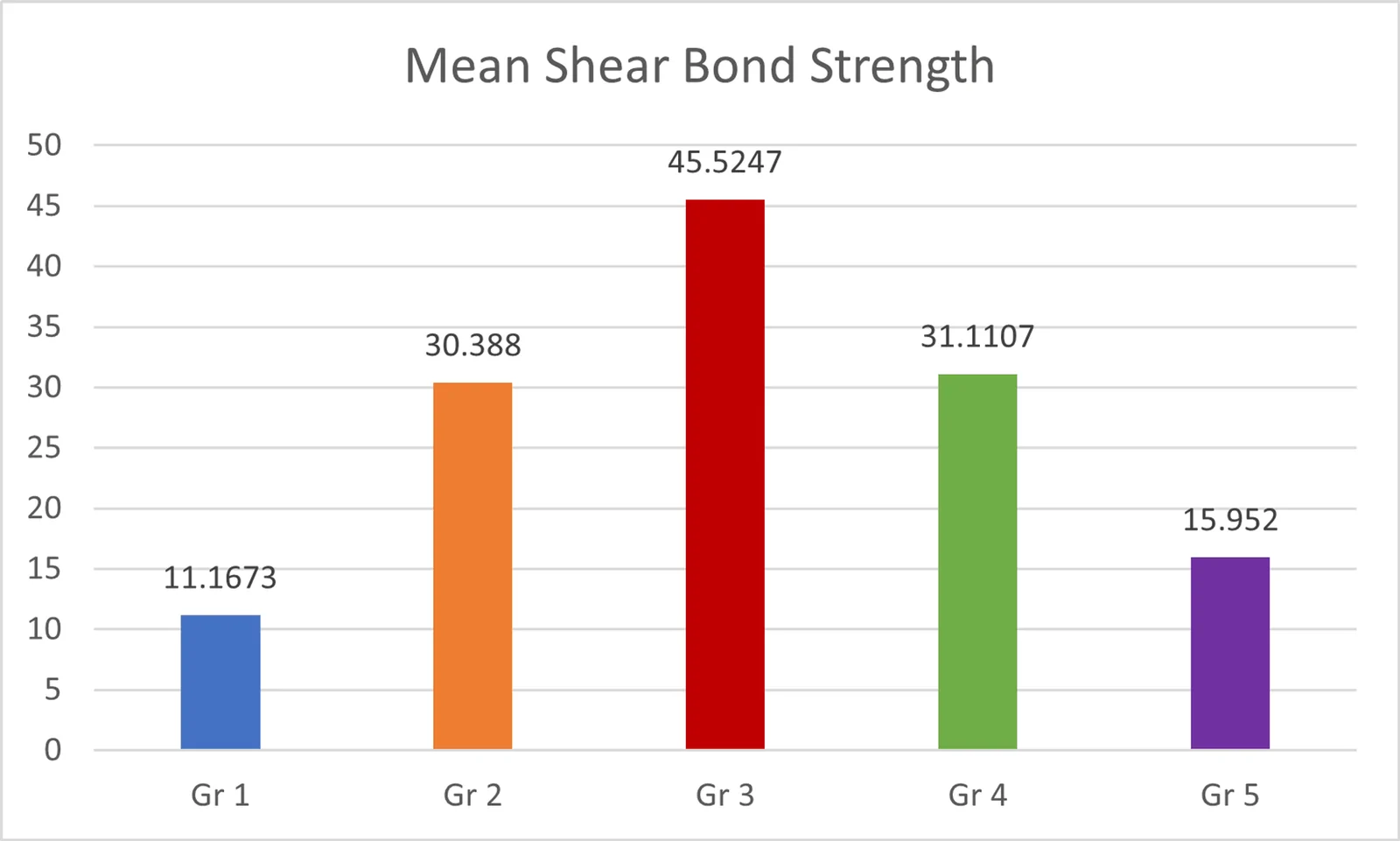
Mean shear bond strength (MPa) of the five experimental groups

The mean SBS values for the five study groups are presented in Table 1 and Figure 6. The highest mean SBS was observed in Group 3 (6.5% grape seed extract containing proanthocyanidin) (45.52 ± 1.37 MPa), whereas the lowest mean SBS was recorded in Group 1 (immediate bonding after bleaching) (11.17 ± 1.66 MPa).

**Table 1:** Descriptive statistics of shear bond strength among the study groups The overall trend in bond strength was the following: Group 3 > Group 4 > Group 2 > Group 5 > Group 1, indicating superior bond strength recovery following proanthocyanidin treatment.

Group	n	Mean ± SD (MPa)	Minimum	Maximum
Group 1 (Immediate Bonding)	15	11.17 ± 1.66	7.95	13.79
Group 2 (Delayed Bonding)	15	30.39 ± 1.51	26.86	32.17
Group 3 (6.5% Proanthocyanidin)	15	45.52 ± 1.37	43.72	48.01
Group 4 (10% Sodium Ascorbate)	15	31.11 ± 1.98	27.50	34.11
Group 5 (10% Selenium)	15	15.95 ± 0.82	14.95	17.25

Groups 2 (delayed bonding after three weeks) and 4 (10% sodium ascorbate) demonstrated comparable mean SBS values of 30.39 ± 1.51 MPa and 31.11 ± 1.98 MPa, respectively. Group 5 (10% selenium) exhibited a mean SBS of 15.95 ± 0.82 MPa, which was greater than that observed in the immediate bonding group but lower than the values recorded in Groups 2-4 (Table 1).

Comparison of SBS among groups

**Figure 7:**
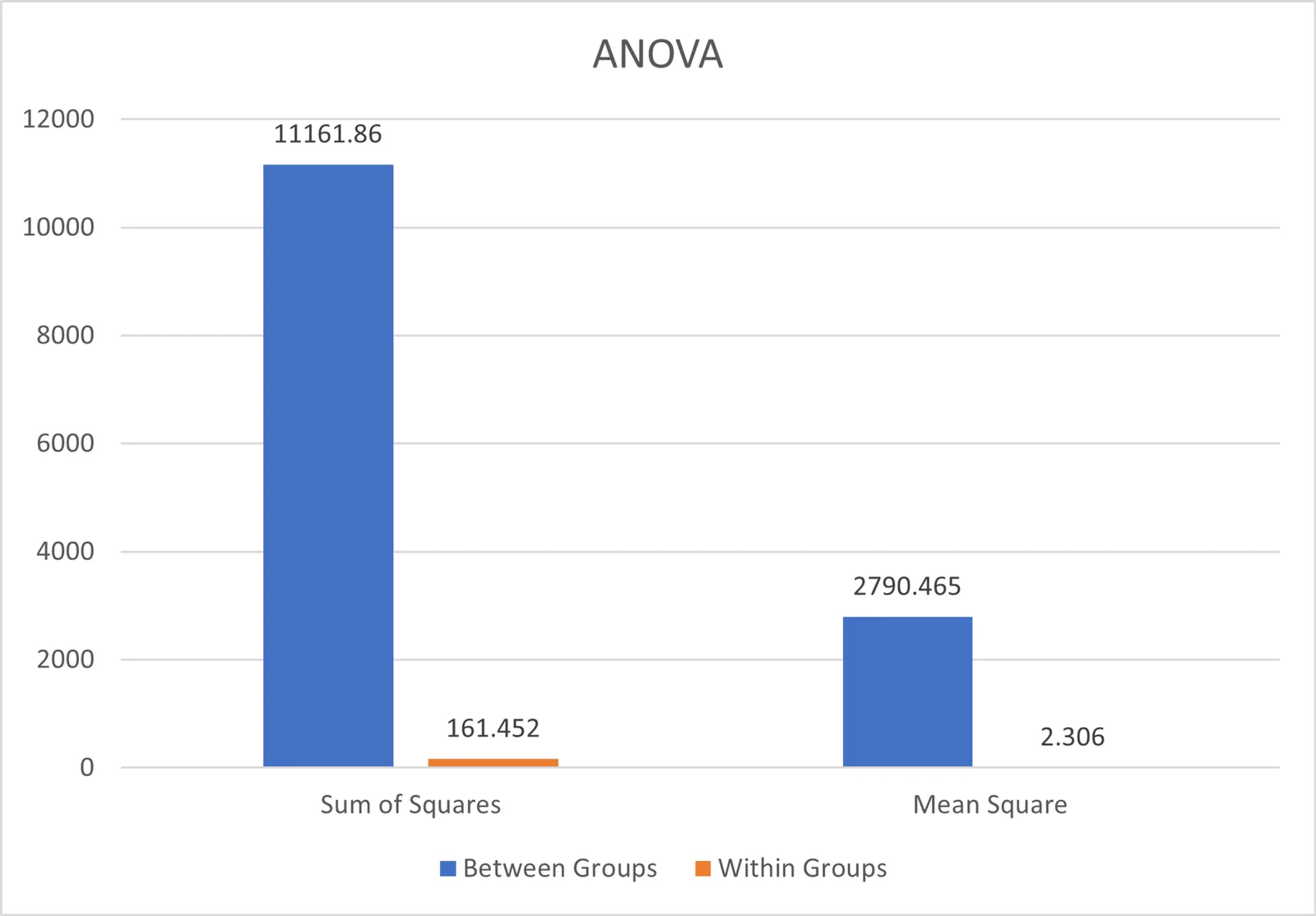
One-way ANOVA demonstrating significant differences in shear bond strength among the study groups ANOVA: analysis of variance

One-way ANOVA demonstrated a highly statistically significant difference in mean SBS among the five groups (F = 1209.846, p < 0.001) as shown in Table 2 and Figure 7.

**Table 2:** One-way ANOVA for comparison of shear bond strength among groups The highly significant p-value indicates that the post-bleaching treatment protocols had a significant effect on the shear bond strength of composite resin bonded to enamel. ANOVA: analysis of variance

Source	Sum of Squares	df	Mean Square	F-value	p-value
Between Groups	11161.860	4	2790.465	1209.846	<0.001
Within Groups	161.452	70	2.306	-	-
Total	11323.31	74	-	-	-

Post hoc analysis

Tukey's HSD test was performed for pairwise comparison of the study groups. Group 3 (6.5% grape seed extract containing proanthocyanidin) exhibited significantly greater SBS values than all other groups (p < 0.001). Group 1 (immediate bonding after bleaching) demonstrated significantly lower SBS values than all remaining groups (p < 0.001).

No statistically significant difference was observed between Group 2 (delayed bonding after three weeks) and Group 4 (10% sodium ascorbate) (p = 0.690), suggesting that sodium ascorbate restored bond strength to a level comparable with delayed bonding. In contrast, Group 5 (10% selenium) demonstrated significantly lower SBS values than Groups 2-4 (p < 0.001), although it showed significantly greater bond strength than Group 1 (p < 0.001).

Key findings

Treatment with antioxidant agents significantly improved the SBS of composite resin to bleached enamel. Among the tested antioxidants, 6.5% grape seed extract containing proanthocyanidin demonstrated the highest bond strength recovery, followed by 10% sodium ascorbate, which exhibited bond strength comparable to delayed bonding. Although selenium improved bond strength relative to immediate bonding, its effectiveness was considerably lower than that of proanthocyanidin and sodium ascorbate. These findings indicate that proanthocyanidin was the most effective antioxidant for reversing the adverse effects of bleaching on enamel bonding.

## Discussion

The present study evaluated the effectiveness of three antioxidant agents - 6.5% grape seed extract containing proanthocyanidin, 10% sodium ascorbate, and 10% selenium - in restoring the SBS of composite resin to enamel following bleaching with 37.5% hydrogen peroxide. The findings demonstrated that antioxidant application significantly improved bond strength when compared with immediate bonding after bleaching. Among the antioxidants tested, proanthocyanidin exhibited the highest bond strength recovery, followed by sodium ascorbate and selenium.

Bleaching with hydrogen peroxide remains one of the most widely used conservative esthetic procedures for the management of tooth discoloration. However, previous investigations have consistently reported a reduction in adhesive bond strength when restorative procedures are performed immediately after bleaching [7]. This reduction is primarily attributed to residual oxygen and peroxide-derived free radicals retained within enamel, which interfere with the free-radical polymerization of adhesive resin systems. These oxidizing species inhibit resin infiltration and polymerization, resulting in compromised bonding at the enamel-resin interface [8].

In the present study, the immediate bonding group demonstrated the lowest mean SBS value (11.17 ± 1.66 MPa). This finding is consistent with previous reports showing significantly reduced bond strength when composite restorations are placed immediately following bleaching. Brock et al. similarly demonstrated that bleaching adversely affects adhesive bond strength because residual oxidizing molecules remain entrapped within enamel and interfere with resin polymerization [9]. These observations support the concept that immediate restorative procedures following bleaching may compromise the longevity and clinical success of adhesive restorations.

The delayed bonding group demonstrated significantly greater SBS values than the immediate bonding group. This improvement may be attributed to the gradual dissipation of residual oxygen from enamel over time, allowing restoration of the normal redox balance and subsequent recovery of adhesive properties. Previous studies have reported that a waiting period of one to three weeks is generally sufficient to restore bond strength to levels comparable to those observed before bleaching. Although effective, delayed bonding prolongs treatment duration and may be inconvenient for patients seeking immediate esthetic rehabilitation.

To overcome the limitations associated with delayed bonding, antioxidant therapy has been proposed as a clinically feasible alternative [10]. Antioxidants neutralize residual free radicals and restore the redox potential of bleached enamel, thereby permitting normal polymerization of adhesive systems. The results of the present study demonstrated that all antioxidant-treated groups exhibited significantly higher SBS values than the immediate bonding group, confirming the effectiveness of antioxidant therapy in reversing the adverse effects of bleaching [11].

Among the antioxidants evaluated, 6.5% grape seed extract containing proanthocyanidin demonstrated the highest mean SBS value (45.52 ± 1.37 MPa), significantly exceeding all other experimental groups. The superior performance of proanthocyanidin may be attributed to its potent antioxidant activity and polyphenolic structure. Oligomeric proanthocyanidin complexes contain multiple hydroxyl groups capable of donating hydrogen atoms to neutralize reactive oxygen species generated during bleaching. In addition, proanthocyanidin has been reported to enhance collagen cross-linking and stabilize the organic matrix of dental tissues, thereby improving substrate integrity and contributing to enhanced bond strength [12].

The results of the current study are in agreement with those previously reported by Vidhya et al., who observed significant improvement in bond strength following application of grape seed extract to bleached enamel [13]. Similarly, Suthakar et al. reported superior bond strength recovery in grape seed extract-treated specimens compared with other antioxidant agents [14]. The enhanced antioxidant activity of proanthocyanidin has been reported to be substantially greater than that of conventional antioxidants such as sodium ascorbate and alpha-tocopherol, which may explain its superior performance in the present investigation.

An important observation in the present study was the exceptionally high SBS value recorded in the proanthocyanidin group. Although this finding highlights the potent efficacy of proanthocyanidin, caution should be exercised when comparing absolute bond strength values with those reported in other studies. Variations in bleaching protocols, antioxidant concentrations, adhesive systems, specimen preparation techniques, storage conditions, and testing methodologies may influence SBS outcomes. Therefore, the superior performance of proanthocyanidin should be interpreted primarily in relation to the other groups evaluated under identical experimental conditions.

The sodium ascorbate group demonstrated a mean SBS value of 31.11 ± 1.98 MPa, which was comparable to the delayed bonding group (30.39 ± 1.51 MPa). Statistical analysis revealed no significant difference between these groups, suggesting that sodium ascorbate effectively restores bond strength to levels comparable with those achieved after a three-week waiting period. Sodium ascorbate functions as a potent reducing agent that neutralizes residual oxygen radicals through electron donation, thereby re-establishing favorable conditions for resin polymerization [15].

These findings are in agreement with those reported by Nari-Ratih and Widyastuti, who demonstrated that sodium ascorbate treatment restored SBS values to levels comparable with delayed bonding and unbleached controls. Due to its low molecular weight, water solubility, and biocompatibility, sodium ascorbate readily penetrates enamel and effectively neutralizes residual oxidants. Consequently, it remains one of the most extensively investigated antioxidant agents for immediate post-bleaching restorative procedures.

The selenium group demonstrated a mean SBS value of 15.95 ± 0.82 MPa. Although selenium significantly improved bond strength compared with immediate bonding after bleaching, its effectiveness was substantially lower than that observed with proanthocyanidin and sodium ascorbate. The comparatively limited efficacy of selenium may be attributed to its indirect antioxidant mechanism. Selenium primarily functions through incorporation into antioxidant enzymes such as glutathione peroxidase, which catalyze the reduction of hydrogen peroxide and other reactive oxygen species. Because the present study was conducted under in vitro conditions, the biological pathways necessary for optimal selenium activity may not have been fully expressed.

Furthermore, selenium appears to possess limited interaction with highly mineralized enamel surfaces, potentially restricting its penetration and retention within the substrate. These factors may explain the lower bond strength values observed in the selenium-treated group. Nevertheless, the findings are partially consistent with those of Halkai et al., who reported improved SBS following selenium application after bleaching. Their study also suggested that combining selenium with other antioxidant agents may further enhance bond strength recovery [16].

Statistical analysis using one-way ANOVA confirmed a highly significant difference among the experimental groups (F = 1209.846, p < 0.001), indicating that the type of post-bleaching treatment exerted a significant influence on bond strength. Subsequent Tukey's post hoc analysis revealed that proanthocyanidin exhibited significantly higher SBS values than all other groups. In contrast, no statistically significant difference was observed between sodium ascorbate treatment and delayed bonding, confirming the ability of sodium ascorbate to serve as a practical alternative to prolonged waiting periods before restoration.

The findings of the present study have important clinical implications. Immediate restoration following bleaching is frequently desired by both clinicians and patients. However, compromised bond strength may adversely affect restoration longevity and clinical success. The results suggest that antioxidant therapy can effectively eliminate the need for prolonged post-bleaching waiting periods. In particular, proanthocyanidin and sodium ascorbate demonstrated substantial potential for immediate chairside application, thereby reducing treatment time and improving patient satisfaction.

However, this study has some limitations, as it was conducted under in vitro conditions and may not completely replicate the clinical oral environment. Factors such as salivary enzymes, thermal cycling, pH fluctuations, masticatory forces, and long-term aging were not evaluated. In addition, only one bleaching agent concentration, one adhesive system, and one composite resin material were investigated. Therefore, caution should be exercised when extrapolating the findings directly to clinical practice.

## Conclusions

Within the limitations of this in vitro study, bleaching significantly reduced the SBS of composite resin when bonding was performed immediately after treatment. Application of antioxidant agents effectively restored bond strength. Among the antioxidants evaluated, 6.5% grape seed extract containing proanthocyanidin demonstrated the highest bond strength recovery, followed by 10% sodium ascorbate, which showed performance comparable to delayed bonding. Selenium exhibited comparatively lower efficacy. These findings suggest that proanthocyanidin may be a promising antioxidant for facilitating immediate restorative procedures following vital bleaching.
